# HHV-6B infection after umbilical cord blood stem cell transplantation with pruritus as the first symptom: HHV-6B infection after transplantation with pruritus

**DOI:** 10.3724/abbs.2023161

**Published:** 2023-08-09

**Authors:** Bin Xu, Yan Jia, Linlin Lv, Lulu Chen, Panpan Cheng, Saisai Ren, Haihui Liu, Min Zhang, Hao Zhang

**Affiliations:** 1 Department of Clinical Medicine Jining Medical University Jining 272000 China; 2 Department of Hematology Affiliated Hospital of Jining Medical University Jining 272000 China

Bone marrow hematopoietic stem cell transplantation (BMT) is one of the most effective treatments for hematological malignancies, and cord blood hematopoietic stem cell transplantation is one of them [1]. Human herpesvirus-6B (HHV-6B) is known as a series of broadly widespread herpes viruses. Primary infection occurs in infants and young children. After infection, the virus is in a state of quiescence, and often reactivates after bone marrow transplantation with a decline in immunity [2]. HHV-6B infection is fatal in bone marrow transplant patients due to the absence of clinical manifestations. In this study, a 15-year-old myeloproliferative neoplasm (MDS/MPN) with eosinophilia patient was infected with HHV-6B 24 days after cord blood hematopoietic stem cell transplantation with pruritus as the initial symptom. The bone marrow diagnosis was shown in Figure 1, and the patient showed an increased proportion of bone marrow blasts, as well as a significant rise in eosinophils. Moreover, abnormalities were observed in the development of the myeloid lineage. The morphology of the patient’s lymphocytes appeared normal, suggesting no significant abnormalities. Lastly, the number and shape of the patient’s platelets were found to be normal. Based on the above results, the patient was finally confirmed diagnosed as MDS/MPN with eosinophilia. After routine chemotherapy and achieving complete remission (CR), hematopoietic stem cell transplantation of cord blood was performed. Chemotherapy before hematopoietic stem cell transplantation was shown in the Supplementary Table S1.

**Figure 1:**
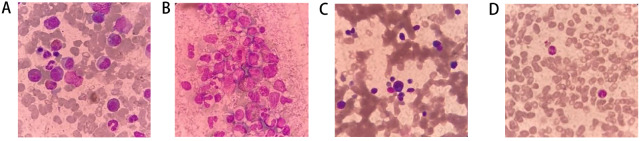
Figure 1 Bone marrow aspiration and bone marrow biopsy image of the patient diagnosed with MDS/MPN at 1 month, and 2 months after transplantation (A) Bone marrow aspirate smear showed increased blasts and an abnormal eosinophilic precursor when MDS/MPN was diagnosed (Wright-Giemsa stain, 1000×). (B) Bone marrow biopsy smears revealed active bone marrow hyperplasia and eosinophils when MDS/MPN was diagnosed (Wright-Giemsa stain, 1000×). (C) Bone marrow smears indicate that the percentage of bone marrow blasts decreased 1 month after UCBT (Wright-Giemsa stain, 1000×). (D) Bone marrow smears suggested that the proportion of bone marrow blasts was lower than that of the first result (Wright-Giemsa stain, 1000×).

The patient underwent an unrelated HLA 9/10 identical unrelated umbilical cord blood transplantation (UCBT) on Dec 21, 2021. The total number of mononuclear cells infused was 19.36×10 ^8^ (3.87×10 ^7^/kg), and the number of CD34+ cells was 13.16×10 ^6^ (2.63×10 ^5^/kg). During transplantation, it is crucial to have an adequate number of mononuclear cells in the graft. For cord blood transplantation, a minimum of 2×10 ^7^ mononuclear cells per kilogram of body weight is typically necessary for successful transplantation. Additionally, CD34+ cells are often identified as hematopoietic stem cells or progenitor cells and have a significant impact on the success rate of transplantation, transplantation-related mortality, and progression-free survival of patients. Although there is no standardized requirement for the amount of CD34+ cells for recipients, previous studies have demonstrated that higher doses of CD34+ cells are associated with improved transplant outcomes. Neutrophil engraftment days and platelet engraftment days were defined as the time from the day of transplantation to the first day of neutrophil/platelet engraftment. The time of neutrophil engraftment was 25 days (Jan 15, 2022) and the time of platelet engraftment was 48 days (Feb 8, 2022). Neutrophil engraftment is on day +25 (Jan 15, 2022) and platelet engraftment is on day +48 (Feb 8, 2022) after transplantation. On day +11 (Jan 1, 2022) after transplantation, IL-6 and other inflammatory factor levels were significantly elevated, indicating a cytokine storm caused by sepsis ( Figure 2). Tocilizumab was applied on Jan 1, 2022‒Jan 5, 2022. After that, the levels of serum inflammatory factors, including IL-6, IL-10, and IL-2, were found to be significantly lower in patients compared to their previous levels. On the +23 day (Jan 13, 2022) after transplantation, the patient complained of persistent pruritus on the back. Physical examination showed no skin manifestations, such as rash, bleeding spots, or other abnormal signs. The next day (Jan 14, 2022), the patient’s skin pruritus was aggravated to intolerable and extended to the whole body. A significant increase in C-reactive protein (CRP) suggests the possibility of infection, and all other results, such as serum creatinine (Scr), total bilirubin (TBIL), and direct bilirubin (DBIL), were unremarkable ( Table 1). We suspect that the patient’s severe pruritus may have been caused by an infection, as an increase in inflammatory factors often precedes an infection. Pruritus was not relieved after clonazepam, gabapentin, pregabalin, chlorphenamine maleate, morphine, and glucocorticoids were administered. The results of Epstein-Barr virus (EBV) DNA and cytomegalovirus (CMV) DNA tests were negative ( Table 2). Therefore, we performed peripheral blood metagenome next-generation sequencing (mNGS) and detected positivity for HHV-6B (160 copies/mL). Moreover, all other bacterial, fungal, and viral species were negative, indicating that the patient’s skin pruritus was related to HHV-6B infection. According to the 2017 European Conference on Infections in Leukemia guidelines [3], the patient was treated with foscarnet, and the pruritus disappeared. We also determined the changes in the inflammatory factors IL-6, IL-10, and IL-2 and the quantitation of HHV-6B DNA copies. The results are shown in Figure 2.

**Figure 2:**
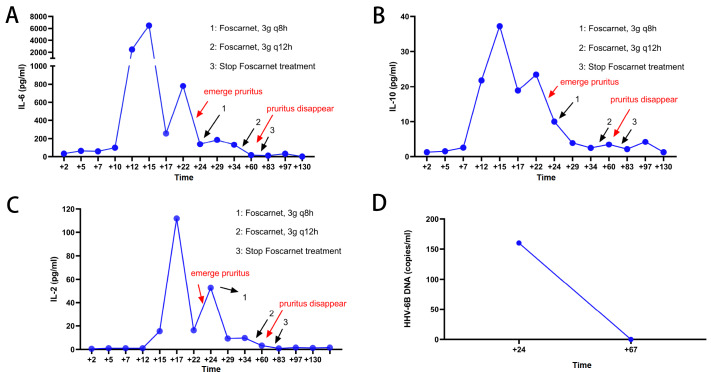
Figure 2 Changes in plasma inflammatory factor and HHV-6B DNA quantitative detection in patients were monitored continuously until they returned to basal levels (A–C) Changes in the levels of the inflammatory factors IL-6, IL-10, and IL-2 in serum. The patient developed pruritus on day +23 day and received treatment with foscarnet (3 g every 8 h) on day +24. Then, we decreased the dose of foscarnet to 3 g every 12 h on day +46. Finally, we stopped the treatment with foscarnet on day +70 after pruritus disappeared. (D) Quantitative detection of HHV-6B DNA was performed on days +24 and +67 to guide the timing and duration of foscarnet.

**Table:** **Table 1** The results of routine blood tests and clinical physiological indicators

	Dec 7, 2021	Dec 21,2021	Jan 1, 2021	Jan 14, 2022	Jan 24, 2022	Feb 19, 2022	Mar 11, 2022
WBC (×10 ^9^/L)	5.75	0.04	0.01	1.25	4.14	5.46	8.72
NEU (%)	8.5	–	–	83.20	78.10	86.90	72.20
LYM (%)	3.3	–	–	3.20	3.60	0.26	5.0
EOS (%)	72.5	–	–	0.80	1.20	0	9.90
BAS (%)	0	–	–	1.60	0.20	0	0.50
Scr (μM)	51.9	4.9	47.8	30.8	35.6	37.6	31.2
ALT (U/L)	5.2	18.9	12.8	60.8	18.9	9.0	39.8
AST (U/L)	12	15.0	11.0	23.0	11.0	12.0	99.0
TBIL (μM)	11.3	7.4	13.6	22.9	39.1	10.0	12.0
DBIL (μM)	3.9	4.4	6.4	8.3	11.2	2.4	1.3
CRP (mg/L)	10.0	22.0	33.0	<1.0	<1.0	15.0	28.6
IL-6 (pg/mL)	–	33.05	97.3	137.62	131.6	16.92	11.65

WBC: white cell counts; NEU: neutrophils; LYM: lymphocytes; EOS: eosinophils; BAS: basophils; Scr: serum creatinine; ALT: alanine aminotransferase; and AST: aspartate aminotransferase.

**Table:** **Table 2** Plasmatic EBV DNA and CMV DNA concentrations for patient determined by quantitative PCR

	Dec 7, 2021	Dec 17, 2021	Dec 24, 2022	Jan 14, 2022	Jan 27, 2022	Feb 5, 2022	Mar 1, 2022	Mar 11, 2022
EBV DNA(copies/mL)	<5.0×10 ^2^	<5.0×10 ^2^	<5.0×10 ^2^	<5.0×10 ^2^	<5.0×10 ^2^	<5.0×10 ^2^	<5.0×10 ^2^	<5.0×10 ^2^
CMV DNA(copies/mL)	<5.0×10 ^2^	<5.0×10 ^2^	<5.0×10 ^2^	<5.0×10 ^2^	<5.0×10 ^2^	<5.0×10 ^2^	<5.0×10 ^2^	<5.0×10 ^2^

Similar to other herpesviruses, HHV-6B enters a chronic or latent infection state in brain tissue, tonsils, and salivary glands after primary infection, and is reactivated in the absence of cellular immunity under conditions such as hematopoietic stem cell transplantation, organ transplantation, and acquired immunodeficiency syndrome (AIDS) [ 4, 5]. Recent discoveries indicated that between 40% and 60% of stem cell transplant recipients experienced HHV-6 infection within 2 to 4 weeks just after transplantation [ 6 , 7]. Primary infection with HHV-6B commonly occurs during childhood. It is usually characterized by roseola infantum, an acute febrile illness that may or may not be accompanied by a rash. In addition to the rash, some children may experience nonspecific symptoms like irritability, cough, and diarrhea. Although rare, there is a possibility for children to develop encephalitis, which can either occur alongside roseola infantum or serve as the initial indication of infection. In adults, primary infection with HHV-6B is extremely rare. However, there have been reports of a mononucleosis-variant syndrome in adults, which is associated with HHV-6B infection and can vary in severity [8]. This syndrome is also accompanied by chronic lymphadenopathy. Due to the rarity of primary infection in adults, it is generally believed that most cases are caused by reactivated infection. In individuals with normal immune function, HHV-6B infection can lead to symptoms such as fever, hepatosplenomegaly, lymphadenopathy, and occasionally encephalitis. The encephalitis can manifest with symptoms like altered levels of consciousness, seizures, psychiatric symptoms, and acute cerebellar ataxia. In immunocompromised populations, particularly transplant recipients, HHV-6B infection can result in various clinical manifestations including fever, rash, pneumonia, hepatitis, encephalitis, and myelosuppression. To date, there have been no reported cases of pruritus following HHV-6B infection in both immunocompetent children and adults, as well as immunocompromised patients. Although risk factors for HHV-6 infection in transplant recipients are unidentified, HHV-6B infection occurs predominantly following umbilical cord blood transplantation (UCBT) compared to other stem cell sources [9]. In this study, we found that HHV-6B infection occurs after an inflammatory storm.

Infection or reactivation of the HHV-6B virus can be fatal to patients who undergo cord blood hematopoietic stem cell transplantation [10]. As the patient’s pruritus emerged, we first thought that it was caused by pathogenic microbiology. Nevertheless, negative peripheral blood cultures, EBV DNA, and CMV DNA quantification ruled out bacterial, fungal, EBV, and CMV infection. Therefore, peripheral blood mNGS was completed, and the results revealed that the patient’s peripheral blood contained HHV-6B DNA (160 copies/mL). Based on peripheral blood mNGS, we suspected that HHV-6B infection was the cause of the patient’s intense pruritus. Foscarnet is an effective anti-HHV-6B agent. Then, we decided to provide foscarnet, and the pruritus healed entirely.

In conclusion, HHV-6B infection with pruritus as the first symptom after UCBT has not been reported. This case indicates that we should strengthen our understanding of HHV-6B infection after UCBT, especially when the symptoms of pruritus after stem cell transplantation and the exclusion of frequent causes should be examined in a timely manner by mNGS. We believe that with the discovery of more instances, the clinical characteristics of HHV-6B infection after UCBT should be further investigated and evaluated.

## Supporting information

23335Supplementary_Table_S1

## Supplementary Data

Supplementary data is available at *Acta Biochimica et Biophysica Sinica* online.
