# Supplementary material for: HHV-6B infection after umbilical cord blood stem cell transplantation with pruritus as the first symptom: HHV-6B infection after transplantation with pruritus
Source: Acta Biochim Biophys Sin (Shanghai). 2023 Aug 9;55(10):1693–6. doi: 10.3724/abbs.2023161 (PMC10577449; doi:10.3724/abbs.2023161)
Supplement: 23335Supplementary_Table_S1 [file 23335Supplementary_Table_S1.pdf]

Supplementary Table S1. The conditioning regimen for the patient

Day	-9	-8	-7	-6	-5	-4	-3	-2	-1	0	+1	+2	+3	+4	+5	+6
Thiotepa	+															
(5 mg/kg)																
DAC	+	+	+	+	+											
(20 mg/m^2)																
Flu			+	+	+	+	+									
(40 mg/m^2)																
Mel								+								
(100 mg/m^2)																
BU		+														
(130 mg/m^2)																
CTX			+	+	+											
(300 mg/m^2)																
CTX													+	+		
(20 mg/kg)																
CSA (1 g/kg)															+	

DAC: decitabine; Flu: fludarabine; Mel: melphalan; BU: busulfan; CTX: cyclophosphamide; CSA: cyclosporine. Height: 1.8 m; weight: 50 kg; BSA: 1.7 m^2.
